# Bipolar disorder in megalencephalic leukoencephalopathy with subcortical cysts: a case report

**DOI:** 10.1186/s12888-020-02750-6

**Published:** 2020-07-03

**Authors:** Masanori Ishikawa, Yoshie Omachi, Noriko Sato, Eiji Nakagawa

**Affiliations:** 1 Department of Social Welfare Service, Faculty of Human Sciences, Mejiro University, 4-31-1 Nakaochiai, Shinjuku-ku, Tokyo, 161-8539 Japan; 2 Department of Psychiatry, National Center Hospital of Neurology and Psychiatry, National Center of Neurology and Psychiatry, Tokyo, 187-8551 Japan; 3 Department of Radiology, National Center Hospital of Neurology and Psychiatry, National Center of Neurology and Psychiatry, Tokyo, 187-8551 Japan; 4 Department of Child Neurology, National Center Hospital of Neurology and Psychiatry, National Center of Neurology and Psychiatry, Tokyo, 187-8551 Japan

**Keywords:** Megalencephalic leukoencephalopathy with subcortical cysts, MLC1, Bipolar II disorder, Catatonia

## Abstract

**Background:**

Megalencephalic leukoencephalopathy with subcortical cysts (MLC), or Van der Knaap disease, is a rare spongiform leukodystrophy that is characterized by macrocephaly, progressive motor dysfunction, and mild mental retardation. It is very rare for mental illness such as psychotic disorders, affective disorders and anxiety disorders to occur in MLC.

**Case presentation:**

A 17-year-old boy was admitted to our hospital after he developed symptoms of depressive state with catatonia after being diagnosed as having MLC with confirmed *MLC1* mutation. His catatonic symptoms were improved with administration of olanzapine and sertraline and he was discharged after 4 months. Several months later, he became hypomanic. He was diagnosed with bipolar II disorder. Mood swings were controlled with the administration of carbamazepine.

**Conclusions:**

This case is the first report of bipolar disorder during the clinical course of MLC. This case indicate the possibility that MLC influences the development of bipolar disorder in MLC, however, further studies involving more patients are required to clarify this.

## Background

Megalencephalic leukoencephalopathy with subcortical cysts (MLC), or Van der Knaap disease, is an autosomal recessive disease accompanied by macrocephaly and neurological disorders. Many cases of MLC are caused by defects in the *MLC1* gene located on chromosome 22q13.33, which encodes a putative CNS membrane protein [1, 2]. Approximately 60% of MLC patients harbor *MLC1* mutations [3]. In this rare spongiform leukodystrophy, macrocephaly develops within the first 12 months of life, motor dysfunction deteriorates with ataxia and spasticity, and there is mild mental retardation [4]. On magnetic resonance imaging (MRI), the brain has a swollen appearance, diffuse abnormality in the cerebral white matter, and subcortical cysts in the anterior temporal and frontoparietal regions. Even though computed tomography (CT) and MRI show these severe abnormalities, clinical symptoms in early childhood are usually mild.

There have been very few reports of mental disorder such as psychotic disorders, affective disorders and anxiety disorders in MLC patients, except for intellectual disorder [3, 5, 6] and our search of the literature failed to identify any case of bipolar disorder during the course of MLC that was similar to a case we encountered. We report here a patient with genetically proven MLC who presented with catatonia, depression, and hypomanic state at 17 years of age and review the literature accordingly. It is first case that affective disorder occurs in a patient with MLC and our case suggests the factors involved in diagnostic and treatment strategies for bipolar disorder in MLC.

## Case presentation

The patient was admitted to our hospital with catatonia at the age of 17 years. He was the first child of healthy parents. His grandmother had been diagnosed with major depression. She did not show a manic state, hypomanic state, epileptic attack, or catatonia. She also had no signs of enlarged head circumference, neurological symptoms, or intellectual disability. Pregnancy, delivery, and the neonatal period were uneventful. He began to walk without support at 15 months, but with a waddling gait. At the age of 10 years he had an episode of seizures and was hospitalized. He was then diagnosed with MLC based on characteristic brain imaging findings and clinical features including macrocephaly, impaired motor function with ataxia and spasticity, and mild mental retardation. Gene analyses revealed an S93L mutation of the *MLC1* gene.

He completed primary education, attending school until 16 years of age, and became depressed at 17 years of age with no precipitating factors. He then quit senior high school. Subsequently, he presented with negativism.

On admission, his height was 157 cm and weight 47.4 kg. Neurological examination revealed macrocephaly with a head circumference of 58 cm and evaluated as being within the 75th percentile [7]. He had spastic paralysis of the left leg and the Babinski reflex was present bilaterally. Gait was unstable with spasticity and ataxia. Routine blood analyses were normal.

Two experienced psychiatrist observed catatonia with diminished interest, insomnia, loss of energy, negativism, mutism, refusal of food, atypical mannerisms, and psychomotor agitation. They were not able to interviewed because of his negativism. His catatonia with negativism persisted for a month after hospitalization. Present history showed depressive episode with loss of interest, insomnia, loss of energy, refusal of food, and psychomotor excitation.

**Fig. 1:**
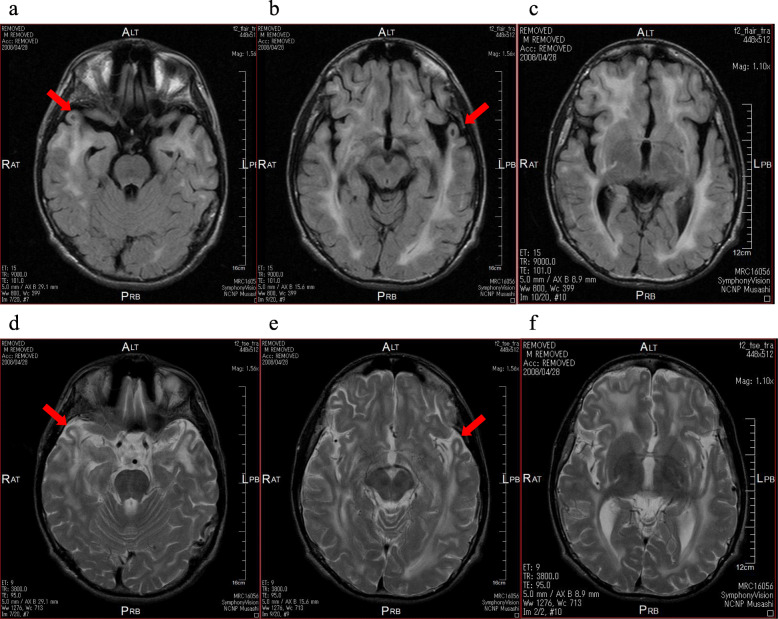
Brain MRI on admission at 17 years of age. **a**, **b**, and **c**: axial FLAIR images. **d**, **e** and **f**: axial T2-weighted images. Diffuse high signal intensity is seen in bilateral cerebral white matter. Frontal and temporal subcortical areas are predominantly involved. Arrows indicate characteristic subcortical cysts in both temporal lobes

Brain Magnetic resonance imaging (MRI) showed diffuse cerebral white matter hyperintensity on T2-weighted and FLAIR images, predominantly in the frontal lobes including the subcortical U-fibers. Subcortical cysts were seen in both temporal poles (Fig. 1 a, b, d, and e). Deep white matter areas, such as corpus callosum, external capsule, and the posterior limb of the internal capsule, also involved. Compared with images obtained at age 10 years, the subcortical cysts were slightly larger and cerebral atrophy had progressed slightly, although diffuse T2 hyperintensity in the cerebral white matter was unchanged.

**Fig. 2:**
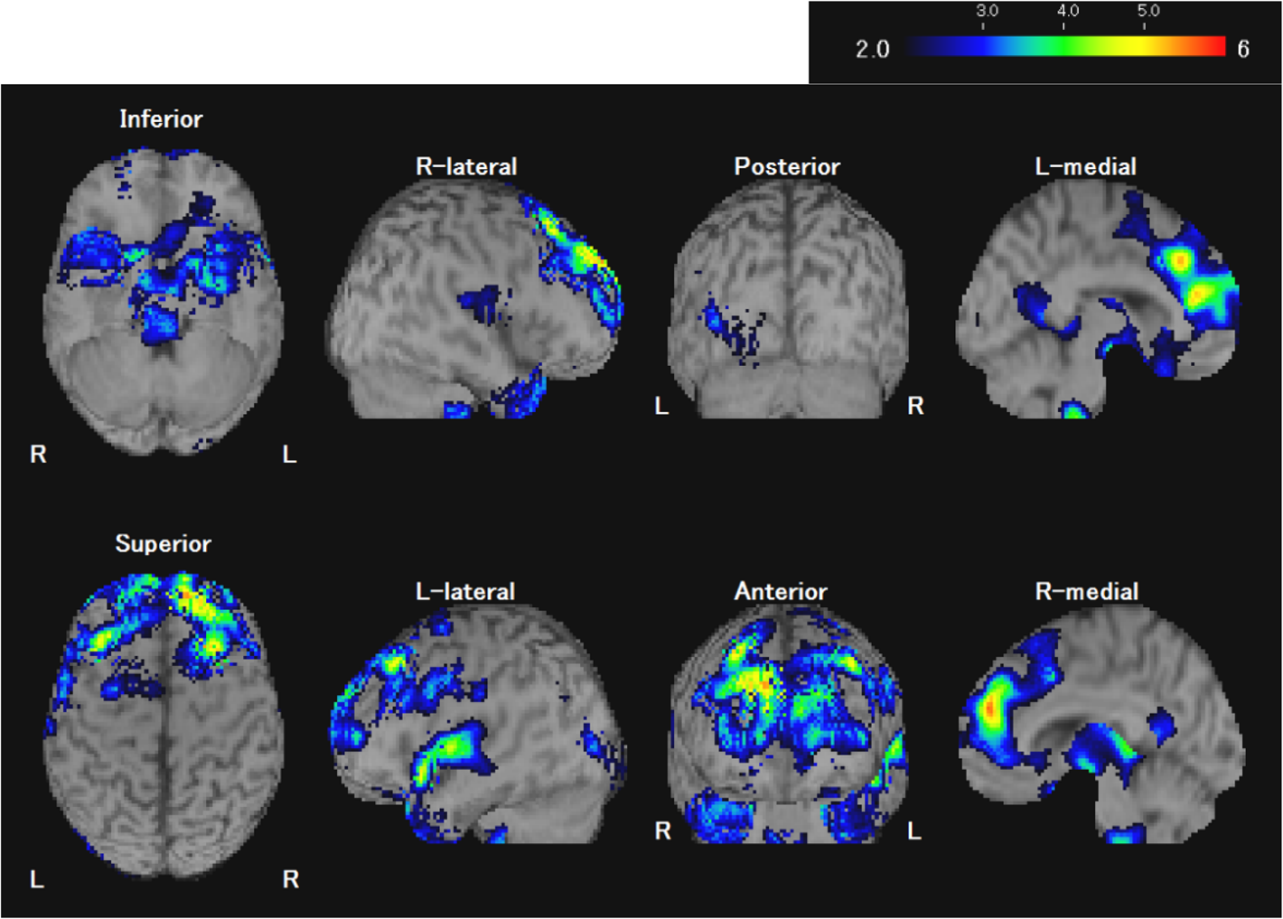
e-ZIS analysis of ^99m^Tc-ECD SPECT at 17 years of age after catatonia. The e-ZIS analysis shows decreased cerebral blood flow in the bilateral prefrontal and anterior temporal cortices

The easy Z-score (eZIS) analysis for ^99m^Tc-ethyl cysteinate dimer-SPECT (^99m^Tc-ECD-SPECT) revealed decreased regional cerebral blood flow in the medial and lateral prefrontal and anterior temporalcortices (Fig. 2). Electroencephalographic (EEG) studies showed a 2 to 3 Hz delta wave independently in the bilateral frontal area during waking and sleep, shown in detail in Additional file 2. Performance IQ was 49, verbal IQ was 62, and total IQ was 52 on the Wechsler Adult Intelligence Scale-III. It was performed after remission of depression with catatonia so that the timing of the examination did not affect the results.

**Fig. 3:**
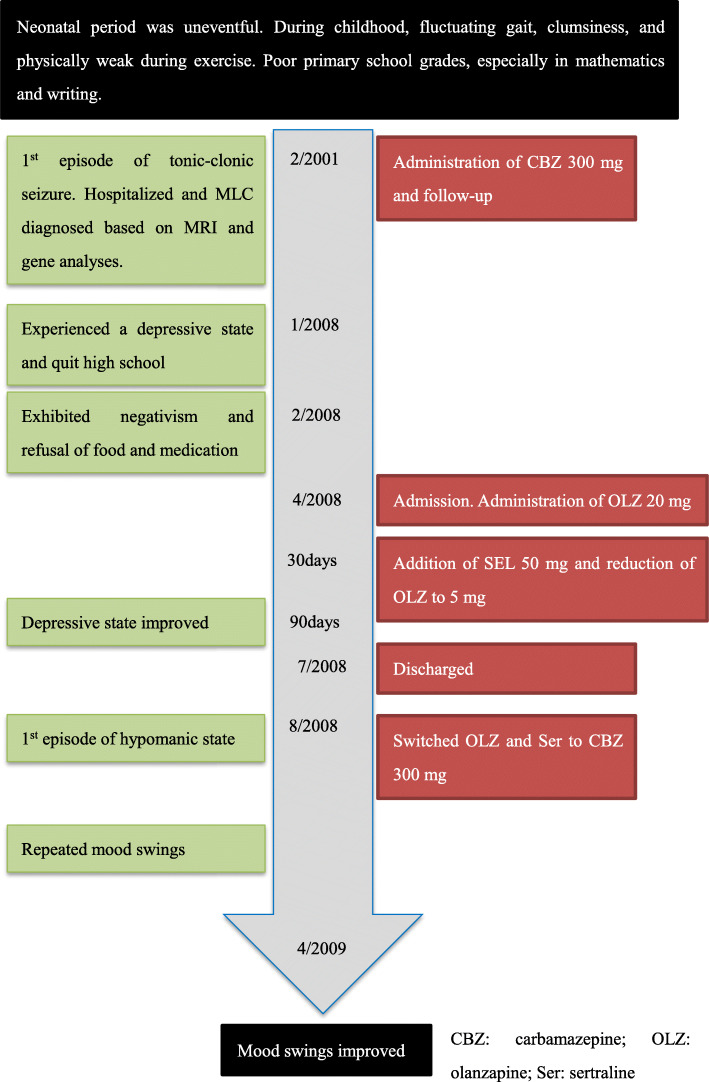
Timeline

We began the administration of olanzapine 20 mg to him after hospitalization. We additioned sertraline 50 mg and reduced olanzapine to 5 mg because the symptoms did not improve after 30 days admission. Catatonia was significantly improved after 90 days admission and he was discharged 4 months later. However, one month after discharge, he developed hypomania with increased energy, decreased need for sleep, excessive talkativeness, and psychomotor agitation. He was diagnosed as having bipolar disorder. We started carbamazepine 300 mg and quitted olanzapine and sertraline although his depression and hypomanic states recurred about every two months. These symptoms were controlled after six months. The timeline is shown in Fig. 3.

## Discussion and conclusions

Our patient’s phenotype was characteristic of MLC with the *MLC1* mutation until adolescence, and diagnosis had been made based on characteristic brain MRI findings and clinical features, such as macrocephaly, motor impairment with ataxia and spasticity, and mild mental retardation. These findings were consistent with the MLC criteria proposed by Van der Knaap who first described the condition in 1995 [4]. In the present patient, the S93L mutation was detected in a homozygous state. This mutation appears to be particularly common in Japanese patients with MLC [8–11].

The present study revealed remarkable symmetrically decreased cerebral blood flow in the bilateral prefrontal, anterior temporal, and parietal cortex on SPECT after catatonia had set in. Sugiura et al. reported similar findings in a SPECT study of MLC [6]. It is possible that these abnormal SPECT findings might be characteristic changes in patients with MLC; further studies involving more patients are needed.

Hypomania and depressive state with catatonia recurred in this patient. During the depressive state, he showed diminished interest, insomnia, loss of energy, refusal of food, and psychomotor excitation. These symptoms were observed on admission by two experienced psychiatrists. These findings were consistent with the clinical features of a major depressive episode as described in the Diagnostic and Statistical Manual for Mental Disorders 5th edition [12] (DSM-5). Furthermore, he exhibited negativism, mutism, and atypical mannerisms, findings also consistent with the syndrome of catatonia in the DSM-5. Together, these findings suggest that the depressive state was a major depressive episode accompanied by catatonia according to the DSM-5. During the hypomanic state, he showed increased activity, decreased need for sleep, excessive talkativeness, and psychomotor agitation. However, the episode was not severe enough to necessitate hospitalization, and there were no psychotic features including catatonia. These symptoms meet the criteria for a hypomanic episode in the DSM-5. In addition, given that a diagnosis of bipolar II disorder requires hypomanic episodes and major depressive episodes, he was also diagnosed as having bipolar II disorder. However, in patients with associated mood disturbance caused by a medical condition, the diagnosis would be “bipolar and related disorders due to another medical condition”.

In addition, pharmacotherapy may have affected psychiatric symptoms. This case showed a hypomanic state while taking sertraline and olanzapine. Previously, there have been two reports of manic status with these drugs [13, 14]. This case exhibits a hypomanic state for several months even when these drugs are not taken. In addition, DSM-5 argues that a full hypomanic episode that emerge during antidepressant treatment including medication but persists at a fully syndromal level beyond the physiological effect of that treatment is sufficient evidence for a hypomanic episode diagnosis [12]. This suggests that the hypomanic state in this case was not caused by these drugs.

The question remains as to whether organic alterations in MLC influence mood. Our initial electronic searches of MEDLINE, PubMed, Cochrane Library, PsycINFO, and EMBASE were conducted in December 2018. We found only three case reports of MLC with mental disorder without mental retardation: depressive state, obsessive-convulsive symptoms, and coma (Table 1) [3, 5, 6]. These findings were not in accord with the features and S93L mutation in the present case. To our knowledge, there is only one report of MLC with mood disorder.

**Table 1:** Comparison of comorbid psychiatric symptoms with MLC in previous reports and present study

Author (Year)	Country (Race)	Onset		Mental retardation	Convulsions	Cysts	Psychiatric symptoms	Gene mutation
		Neurological symptoms	Psychiatric symptoms					
Blattner [3] (2003)	UK	Early childhood	Late 20s	Mild form 9 years old	+	Bilateral temporal lobe	Depressive state, agitation	Not investigated
	(Caucasian)							
Bugiani [5] (2003)	Italy	6 years	12 years		+	Bilateral temporal lobe	Coma	GT to AT^*1^
	(NA)							
Sugiura [6] (2006)	Japan	Early childhood	12 years	Mild	+	Bilateral frontal and temporal lobes	Obsessive-compulsive symptoms	Ser280 → Leu
	(Asian)							
Present case	Japan (Asian)	Early childhood	17 years	Mild	+	Left anterior temporal lobe	Mood swings, catatonia	S93L

*1: IVS5 + 1, G > A. *2: c.1026–1031 delTGCTGC. NA: not available

Our patient had a family history of mood disorder. His grandmother had been diagnosed with major depressive disorder. She did not show any symptoms of bipolar disorder or catatonic symptom. She also had no symptoms related to MLC. It was well established in twin, adoption, and family studies that genetic factors contribute to bipolar disorder [15]; however, we did not perform any genetic analysis of his family members. The possibility that bipolar disorder and MLC are not comorbidities but rather the mood disorder is due to organic changes in the brain caused by MLC cannot be ruled out.

It is also possible that the mutation influenced the mood disorder. This patient had S93L mutation in *MLC1*. Verma et al. explored the association of this gene with bipolar disorder in individuals with these disorders and suggested that *MLC1* was a susceptibility gene [16]. These findings provide support to the notion that *MCL1* mutation may be associated with the onset of bipolar disorder.

Some limitations exist in this case. First, it was difficult to determine whether the bipolar disorder was an organic mood disorder caused by MLC or simply a comorbidity of MLC. Second, because we did not explore genetic analysis of his family members, we cannot clarify whether the mutation influenced the mood disorder. Finally, because there are no similar cases of bipolar disorder during the course of MLC, we have insufficient information to determine whether these symptoms are characteristic of the course of bipolar disorder with MLC. These limitations need to be resolved by further studies involving more patients.

In conclusion, this is the first report of a patient with bipolar disorder during the course of MLC. The course of MLC was typical until adolescence. He then developed depressive state with catatonia at age 17 years and was admitted to our hospital. After discharge, he presented with mood swings including depressive state and hypomania. Mood was stabilized by treatment with carbamazepine. *MLC1* is suspected to be involved in his bipolar disorder and catatonic schizophrenia.

It is unclear whether MLC and bipolar disorder co-occur by chance or are causally related. Taken together, the results indicate that MLC may influence the onset of bipolar disorder. Further studies are needed to better understand the exact impact of organic changes and bipolar disorder in MLC.

## Supplementary information

**Additional file 1.** EEG studies before and after catatonia. a: 10 days after admission during catatonia. b: 27 days after admission with catatonia improved. Each EEG shows 2 to 3 Hz delta waves independently in the bilateral frontal area during waking and sleep with or without catatonia.

**Additional file 2.**^99m^Tc-ECD brain SPECT at 17 years of age after catatonia. Conventional display of Tc-99 m ECD brain perfusion SPECT shows hypoperfusion in the bilateral prefrontal cortex.

## Data Availability

All data generated and analyzed during this study are included in this published article.

## Abbreviations

DSM-5: Diagnostic and Statistical Manual for Mental Disorders, fifth edition
ECD: ^99m^Tc-ethyl cysteinate dimer
EEG: Electroencephalography
FLAIR: Fluid-attenuated inversion recovery
MLC: Megalencephalic leukoencephalopathy with subcortical cysts
MRI: Cerebral magnetic resonance imaging
SPECT: Single-photon emission computed tomography
