# Supplementary material for: Bipolar disorder in megalencephalic leukoencephalopathy with subcortical cysts: a case report
Source: BMC Psychiatry. 2020 Jul 3;20:349. doi: 10.1186/s12888-020-02750-6 (PMC7333431; doi:10.1186/s12888-020-02750-6)
Supplement: Supplementary file 1 — Additional file 1. EEG studies before and after catatonia. a: 10 days after admission during catatonia. b: 27 days after admission with catatonia improved. Each EEG shows 2 to 3 Hz delta waves independently in the bilateral frontal area during waking and sleep with or without catatonia. [file 12888_2020_2750_MOESM1_ESM.ppt]

## Slide 1
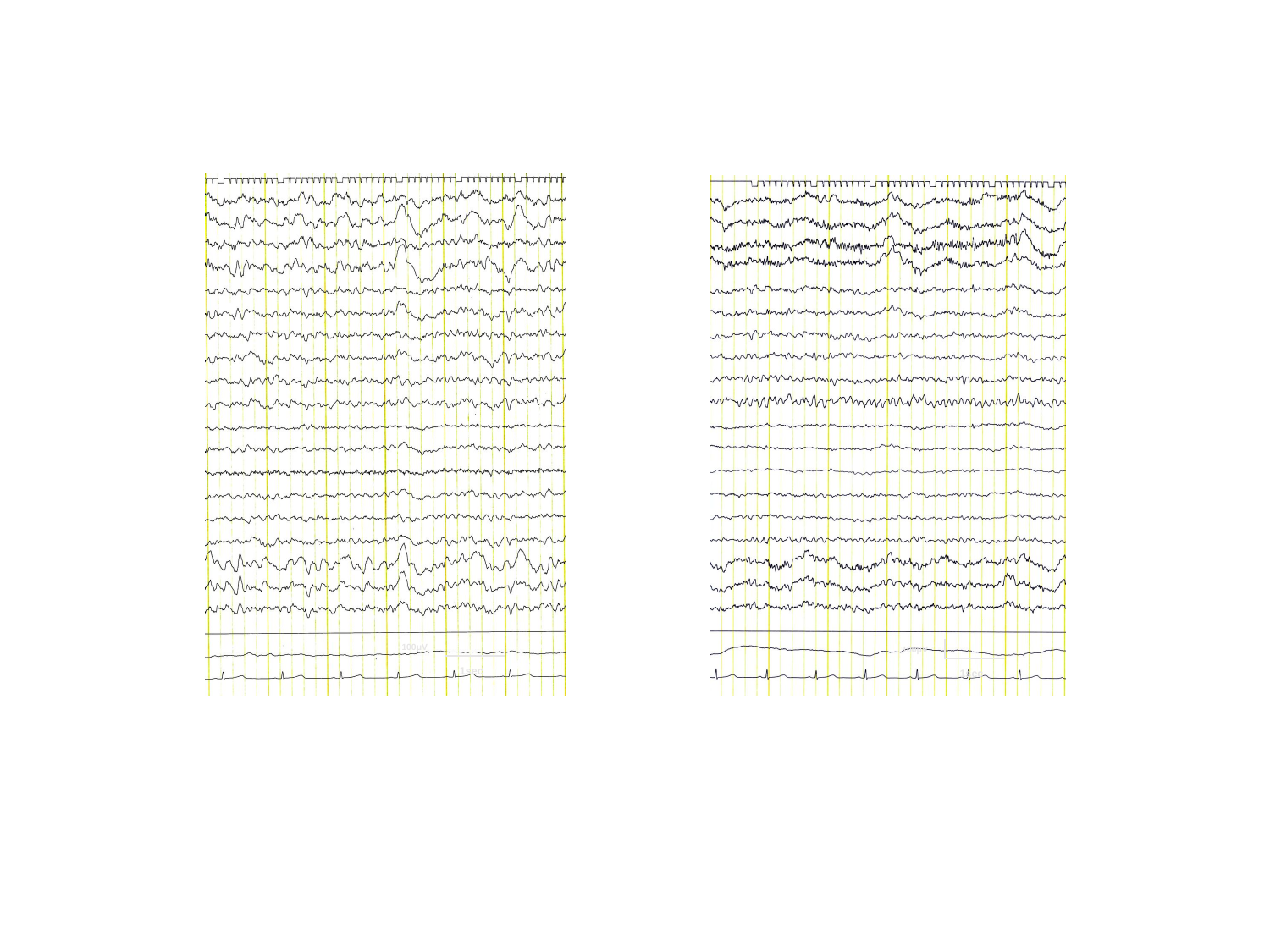

a
b
Fp1-A1
Fp2-A2
F3-A1
F4-A2
C3-A1
C4-A2
P3-A1
P4-A2
O1-A1
O2-A2
F7-A1
F8-A2
T3-A1
T4-A2
T5-A1
T6-A2
Fz-AV
Cz-AV
Pz-AV
EOG
ECG
Fp1-A1
Fp2-A2
F3-A1
F4-A2
C3-A1
C4-A2
P3-A1
P4-A2
O1-A1
O2-A2
F7-A1
F8-A2
T3-A1
T4-A2
T5-A1
T6-A2
Fz-AV
Cz-AV
Pz-AV
EOG
ECG
100μV
1sec
100μV
1sec
