# Supplementary figures and images for: Bipolar disorder in megalencephalic leukoencephalopathy with subcortical cysts: a case report
Source: BMC Psychiatry. 2020 Jul 3;20:349. doi: 10.1186/s12888-020-02750-6 (PMC7333431; doi:10.1186/s12888-020-02750-6)

## Slide 1
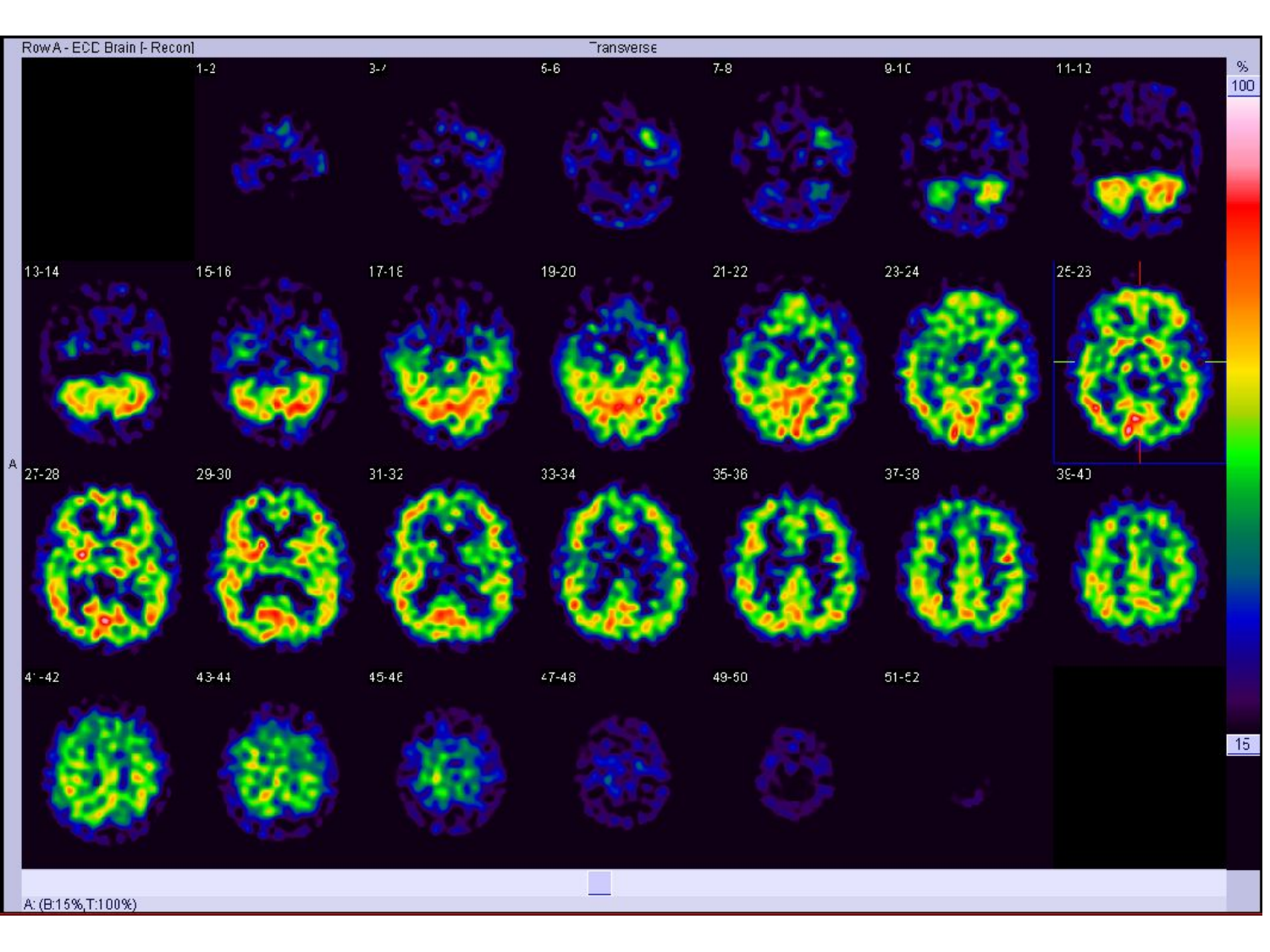

#

Supplement: Supplementary file 2 — Additional file 2.99mTc-ECD brain SPECT at 17 years of age after catatonia. Conventional display of Tc-99 m ECD brain perfusion SPECT shows hypoperfusion in the bilateral prefrontal cortex. [file 12888_2020_2750_MOESM2_ESM.pptx]
